# The Medical Conscience

**Published:** 1906-12-15

**Authors:** 


### The Medical Conscience.

It not infrequently arises in the relations of the doctor with his patients that perplexing problems present themselves to his mind as to how far he is justified in withholding the truth, or in adopting subterfuges of manner or speech with the intent to deceive. Owing to the lack of technical knowledge on the part of patients it is often difficult or impossible to convey to their minds the true condition of things, and by attempting to do so, serious risk may be incurred of giving them a much exaggerated impression of the seriousness of their condition. In order to avoid this the doctor rather chooses the course of using such expressions as will allay any extreme fears that may be already entertained in the mind of the patient; even in cases in which the doctor believes he is dealing with a definitely fatal disease, it is unnecessary, nay it would be brutal and inhuman, to inform the patient of his condition in so many words. Any such expression of opinion can always be tempered with an element of hope. Even cases of cancer are known occasionally to terminate favourably, or the diagnosis may after all be incorrect. Again, it is now very generally recognised that in many morbid conditions, especially in those of a nervous or neurasthenic order, suggestion forms a most useful therapeutic measure, and this frequently involves methods of deception. In all cases it is essentially the interests of the patients themselves that the doctor has to consider. Hence in their interests, and in their interests alone, is he justified in adopting methods of speech or action which may mislead or deceive. On the other hand, there are cases in which a too open expression of opinion in regard to the patient's condition may be avoided for fear of the immediate dismissal of the doctor's services. Take, for instance, those cases in which the patient is undermining his constitution by some evil habit, such as alcoholic excess. We have no hesitation in saying that a doctor will not further the interests of his patient, any more than his own interests, by making a frontal attack upon pernicious habits. Aggressive plain speaking will effectually drive the patient away to another more discreet doctor, but will have no effect upon his consumption of liquor. It is only by securing his confidence, by careful and diplomatic treatment, that one can hope to influence such an individual.

More serious questions touching the medical conscience arise in regard to the actual medical or surgical treatment of the patient. It is the undoubted duty of the doctor not only to treat his patient to the best of his ability and skill, but to supplement that skill by further assistance in any respect in which he may feel himself not fully qualified. It is frequently the privilege of a doctor to have the unbounded confidence of his patient, and it is the more incumbent upon him not to abuse that confidence by undertaking tasks which require a greater skill than his experience has enabled him to attain. If that greater skill is not to hand, he must needs depend upon his own resources, but he is not justified in sacrificing the patient's interests, and possibly his life, to mere professional pride or zeal as by undertaking operations which should properly be committed to the more technically skilled operating surgeon. But it is the pernicious principle involved in the treatment of patients by contract that should weigh most heavily upon the conscience of the medical profession. Under this system doctors undertake to attend patients at a rate of remuneration which leaves no margin for the cost of drugs such as may be most essential for the proper cure of a disease, and the recovery of a patient. It is perhaps hardly to be wondered at under these circumstances if such drugs are withheld, even possibly to the serious detriment of the patient. The doctor must live, but it is often impossible to earn a decent livelihood under these conditions and at the same time to deal honestly with the patients. Unfortunately these club patients are wholly ignorant of the actual facts. They are led to believe that by their paltry subscriptions they are entitled to receive proper and efficacious medical treatment. They suffer for their ignorance by inefficient treatment, and the doctor suffers by being compelled, either to do work at a loss, or to conduct his practice on lines contrary to his professional obligations. There is only one solution to this unfortunate state of affairs; it is to make it known to the public that medical men cannot in the nature of things carry out these contracts at inadequate rates of remuneration. If the public once grasp the facts of the case they will learn to estimate the care of their health at a proper value. It is much to be deplored that such commercial considerations should enter into the professional dealings of the medical man. But it is even more deplorable, that, they should ever directly conflict with his professional duties and moral obligations to his patients.

